# A Comparative Study of the Safety and Efficacy Between Angiotensin-Converting Enzyme Inhibitors and Angiotensin Receptor Blockers on the Management of Hypertension: A Systematic Review

**DOI:** 10.7759/cureus.54311

**Published:** 2024-02-16

**Authors:** Tariladei S Peresuodei, Abhishek Gill, Chijioke Orji, Maiss Reghefaoui, Michell Susan Saavedra Palacios, Tuheen Sankar Nath

**Affiliations:** 1 Internal Medicine, California Institute of Behavioral Neurosciences & Psychology, Fairfield, USA; 2 Orthopedics, California Institute of Behavioral Neurosciences & Psychology, Fairfield, USA; 3 Internal Medicine, University of Debrecen, Debrecen, HUN; 4 College of Medicine, University of Cuenca, Cuenca, ECU; 5 Surgical Oncology, Tata Medical Center, Kolkata, IND

**Keywords:** angiotensin-converting enzyme inhibitors, lisinopril, enalapril, benazepril, candesartan, irbesartan, losartan, angiotensin receptor blockers, high blood pressure, hypertension

## Abstract

Renin-angiotensin-aldosterone system (RAAS) inhibitors, including angiotensin-converting enzyme inhibitors (ACEIs) and angiotensin receptor blockers (ARBs), are commonly used in the management of hypertension. High blood pressure is a vital risk factor for cardiovascular disease. This study aims to establish any significant difference in using ACEIs and ARBs in managing hypertension. We followed the Preferred Reporting Items for Systematic Reviews and Meta-Analyses (PRISMA) guidelines to conduct this systematic review. We searched PubMed, MEDLINE, and ScienceDirect for articles published in the last 20 years (2003 to 2023). Our search was last done on the 27th of June, 2023. Following the initial search, 8,313 articles were found on PubMed. After screening the articles selected from the databases, 10 articles examining 1,621,445 patients were selected for the final study. Three articles were identified that compared ACEI and ARB in their capacity to lower blood pressure. Six articles compared both medications' capacity to reduce cardiovascular events and mortality. Five articles were identified that compared both classes of drugs for adverse effects. This study was made to determine whether or not there is a difference between the use of ACEIs and ARBs in the treatment of hypertension. The study showed that both ACEIs and ARBs are similar in their efficacy in lowering blood pressure. However, ACEI was revealed to be superior to ARB in reducing cardiovascular events and all-cause mortality. ARB was shown to be better tolerated by patients than ACEI.

## Introduction and background

Introduction

"An estimated 1.28 billion adults aged 30-79 years worldwide have hypertension, most (two-thirds) living in low- and middle-income countries"-World Health Organization [1]. Renin-angiotensin-aldosterone system (RAAS) inhibitors, including angiotensin-converting enzyme inhibitors (ACEIs) and angiotensin receptor blockers (ARBs), are widely used in the management of hypertension [2-5]. While both ACEIs and ARBs effectively control blood pressure, they exert their effects at different sites. ACEIs inhibit the conversion of angiotensin-1 to the active angiotensin-2, whereas ARBs competitively bind to angiotensin-2 receptors, thereby inactivating it [6-8]. High blood pressure is a vital risk factor for cardiovascular disease [9,10]. Although both classes of antihypertensives are used for managing hypertension, only a few studies have compared both classes of medications for safety and efficacy in lowering blood pressure and preventing cardiovascular outcomes [11-14]. This study aims to establish any significant difference in using ACEIs and ARBs in managing hypertension [15-20]. This would help clinicians better decide which class of antihypertensive is superior in controlling high blood pressure and preventing mortality from adverse cardiovascular events and other related causes [21-24].

Method 

We used the Preferred Reporting Items for Systematic Reviews and Meta-Analyses (PRISMA) guidelines to conduct this systematic review [25].

Inclusion Criteria

Only systematic review articles were included in this study. Only studies published in English and within the last 20 years (2003 to 2023) were included in our study. We only included human studies. We only included studies on adult populations (18 years and above). We included studies that compared ACEIs against ARBs. In this paper, we included one study that examined the efficacy of ACEs against placebo and another that examined ARBs against placebo.

Exclusion Criteria

We excluded articles that combined ACEIs and ARBs as a joint group of medication (RAAS inhibitors) compared to other antihypertensives. Studies about children (below 18 years of age) were excluded. Animal studies were excluded.

Data Sources

We searched PubMed, MEDLINE, and ScienceDirect for articles published in the last 20 years (2003 to 2023). Our search was done between May and June, 2023. The date of our last search is the 27th of June, 2023. This is represented in Table 1.

**Table 1:** Databases used in this article Mesh: Medical Subject Headings

Database	Search strategy	Papers identified
PubMed/MEDLINE​	MeSH​	8,313 ​
PubMed	Regular keyword​	282​
ScienceDirect​	Regular keyword	3,239 (2021-2023)​

Search Strategy 

Using Boolean "AND" and "OR", we combined the following keywords: hypertension, high blood pressure, angiotensin-converting enzyme inhibitor, benazepril, captopril, enalapril, fosinopril, lisinopril, moexipril, perindopril, quinapril, ramipril, trandolapril, angiotensin receptor blockers, losartan, valsartan, irbesartan, and candesartan. We used the Medical Subject Headings (MeSH) search strategy to generate the following keywords and combined them in our search of PubMed/MEDLINE. The search was restricted to MeSH, drug therapy, therapy, toxicity, therapeutic use, and adverse effects. This is shown in Table 2.

**Table 2:** Search strategy used in this article

Database: PubMed/MEDLINE Date: 27/06/2023	Search words	Number of entries
#1	Hypertension OR high blood pressure OR ( "hypertension/drug therapy"[Majr] OR "hypertension/therapy"[Majr] )	757,333
#2	Angiotensin receptor blockers OR losartan OR valsartan OR irbesartan OR candesartan OR ( "angiotensin receptor antagonists/adverse effects"[Majr] OR "angiotensin receptor antagonists/therapeutic use"[Majr] OR "angiotensin receptor antagonists/toxicity"[Majr] )	45,410
#3	Angiotensin-converting enzyme inhibitors OR benazepril OR captopril OR enalapril OR fosinopril OR lisinopril OR moexipril OR perindopril OR quinapril OR ramipril OR trandolapril OR ( "angiotensin-converting enzyme inhibitors/adverse effects"[Majr] OR "angiotensin-converting enzyme inhibitors/therapeutic use"[Majr] OR "angiotensin-converting enzyme inhibitors/toxicity"[Majr] )	64,451
#4	#1 AND #2 AND #3	8,313

Screening

Two authors searched the databases independently to select articles that met the inclusion and exclusion criteria. Where there were disagreements, a third author was included to resolve this. We checked the titles of the articles and the abstract to select articles that would fit the goal of this study. Twenty-five articles were selected after screening. 

Risk of Bias

Two authors were involved in the appraisal of the selected articles for quality. Both authors agreed upon 10 articles that met this study's goal. The assessment of multiple systematic reviews (AMSTAR) checklist for systematic reviews was used, and only medium- and high-quality studies were included [26]. Quality assessment is summarized in Table 3:

**Table 3:** Summary of risk of bias and quality assessment AMSTAR: assessment of multiple systematic reviews; PICO: population, intervention, comparators, outcomes; RoB: risk of bias; RCTs: randomized controlled trials

AMSTAR	Li et al. (2014) [2]	Salvador et al. (2017) [6]	Xie et al. (2018) [9]	Powers et al. (2012) [11]	Matchar et al. (2008) [12]	Wang et al. (2018) [27]	lv et al. (2018) [28]	Chaugai et al. (2016) [29]	Heran et al. (2008) [30]	Heran et al. (2008) [31]
Did the research questions and inclusion criteria for the review include the components of PICO?	YES	YES	YES	YES	YES	YES	YES	YES	YES	YES
Did the report of the review contain an explicit statement that the review methods were established prior to the conduct of the review, and did the report justify any significant deviations from the protocol?	YES	NO	YES	YES	YES	NO	YES	NO	YES	YES
Did the review authors explain their selection of the study designs for inclusion in the review?	YES	YES	YES	YES	YES	YES	YES	YES	YES	YES
Did the review authors use a comprehensive literature search strategy?	YES	YES	YES	YES	YES	YES	YES	YES	YES	YES
Did the review authors perform study selection in duplicate?	YES	YES	YES	YES	YES	YES	YES	YES	YES	YES
Did the review authors perform data extraction in duplicate?	YES	YES	YES	YES	YES	YES	YES	YES	YES	YES
Did the review authors provide a list of excluded studies and justify the exclusions?	YES	NO	NO	NO	NO	NO	YES	NO	YES	YES
Did the review authors describe the included studies in adequate detail?	YES	YES	YES	YES	YES	YES	YES	YES	YES	YES
Did the review authors use a satisfactory technique for assessing the risk of bias (RoB) in individual studies that were included in the review?	YES	YES	YES	YES	YES	YES	YES	YES	YES	YES
Did the review authors report on the sources of funding for the studies included in the review?	YES	YES	YES	YES	YES	YES	YES	YES	YES	YES
If meta-analysis was performed, did the review authors use appropriate methods for statistical combination of results? RCTs	UNCLEAR	YES	YES	YES	YES	UNCLEAR	YES	YES	UNCLEAR	UNCLEAR
If meta-analysis was performed, did the review authors assess the potential impact of RoB in individual studies on the results of the meta-analysis or other evidence synthesis?	UNCLEAR	YES	YES	YES	YES	UNCLEAR	YES	YES	UNCLEAR	UNCLEAR
Did the review authors account for RoB in individual studies when interpreting/ discussing the results of the review?	YES	YES	YES	YES	YES	YES	YES	YES	YES	YES
Did the review authors provide a satisfactory explanation for, and discussion of, any heterogeneity observed in the results of the review?	YES	YES	YES	YES	YES	YES	YES	YES	YES	YES
If they performed quantitative synthesis, did the review authors carry out an adequate investigation of publication bias (small study bias) and discuss its likely impact on the results of the review?	YES	YES	YES	YES	NO	YES	YES	YES	YES	YES
Did the review authors report any potential sources of conflict of interest, including any funding they received for conducting the review?	YES	YES	YES	YES	YES	YES	YES	YES	YES	YES

Risk of Bias Across Studies

We only selected free articles, while those requiring payment for full access were not selected, even though some met our inclusion criteria. Only systematic reviews and systematic reviews/meta-analyses were included in our study.

Results

**Figure 1:**
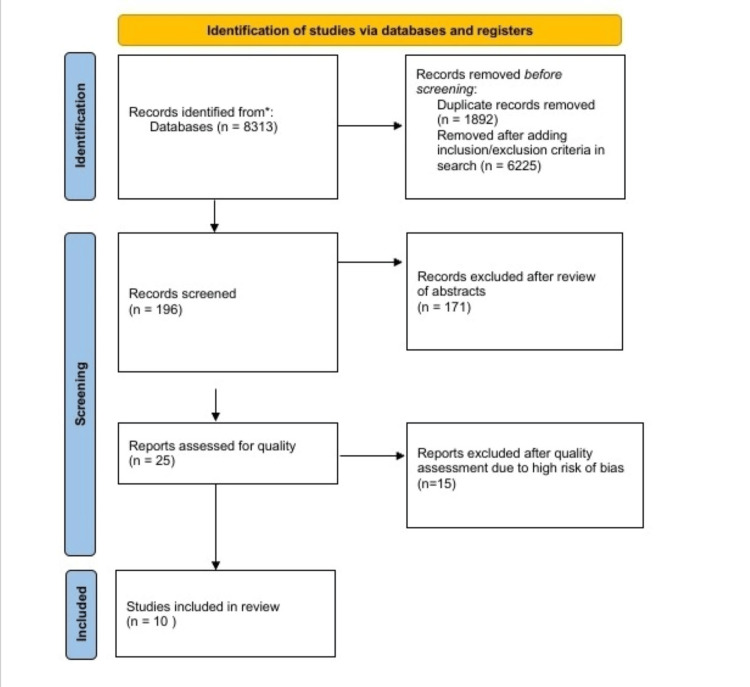
Flow diagram of search strategy and article selection

After the initial search, 8,313 articles were found on PubMed, and 1,892 duplicates were found and removed. A total of 196 articles were left after applying some inclusion and exclusion criteria in the search. After thoroughly applying the criteria for inclusion, only 25 articles were selected. These articles were assessed for quality, after which 10 articles examining a combined total of 1,621,445 patients were selected for the final study. Three articles were identified that compared ACEI and ARB in their capacity to lower blood pressure. Six articles compared both medications' capacity to reduce cardiovascular events and mortality. Five articles were identified that compared both classes of drugs for adverse effects. The search and screening is represented in Figure 1.

## Review

Discussion

This study aims to compare the safety and efficacy of ACEIs and ARBs in managing hypertension. 

Efficacy in Blood Pressure Control

In a systematic review by Powers et al. carried out in 2012 that compared the effectiveness of ACEI, ARB, and direct renin inhibitors (DRIs) on the management of essential hypertension, 33,611 patients were evaluated for blood pressure [11]. The study was an update on a systematic review initially done in 2007. According to the study, there was no statistical difference in blood pressure control when using either ARBs or ACEIs. When either an ACEI was used alone in therapy or an ARB was used alone, no significant difference was found in their efficacy in reducing blood pressure [11]. Another systematic review conducted by Li et al. in 2014 that compared ARBs to ACEIs in managing hypertension sampled 11,007 patients who were followed up for at least one year [2]. Matchar et al. (2008) also conducted a systematic review that compared ACEI and ARB in managing hypertension. The study sampled 13,532 participants for blood pressure and was of good quality [12]. All three studies showed no clinical significance in the difference between the use of ACEIs and ARBs in managing essential hypertension [2,11,12]. This is summarized in Table 4.

**Table 4:** ACEI vs ARB in reducing blood pressure ACEI: Angiotensin-converting enzyme inhibitor; ARB: angiotensin receptor blocker

Author and year of publication	Intervention/drug of study	Number of patients	Type of study	Result/conclusion
Li et al. (2014) [2]	Blood pressure/(ACEI vs ARB)	11,007	Systematic review	There is no significant difference between ACEI and ARB in managing hypertension
Powers et al. (2012) [11]	Blood pressure/(ACEI vs ARB)	798,810 (33,611 for blood pressure)	Systematic review	There is no statistical difference between ACEI and ARB
Matchar et al. (2008) [12]	Blood pressure/(ACEI vs ARB)	345,594 (13,532 for blood pressure)	Systematic review	There is no significant difference between ACEI and ARB in managing hypertension

Mortality and Cardiovascular Outcomes

In a systematic review and meta-analysis comparing ACEIs and ARBs in the reduction of mortality sampling 73,761 participants conducted by Salvador et al. in 2017, ACEIs were shown to have higher efficacy in reducing mortality due to cardiovascular events and all-cause mortality when compared to ARBs [6]. In a systematic review and meta-analysis by Xie et al. in 2018 that studied the effects of antihypertensives and secondary prevention of cardiovascular diseases, 143,095 participants were examined [9]. It was revealed that while ACEIs were more effective in reducing all-cause mortality and cardiovascular events, ARBs were shown to reduce the risk of combined events. A systematic review by Powers et al., conducted in 2012, compared the efficacy of ACEI, ARB, and DRI in managing essential hypertension [11]. The study included 798,810 participants who were examined for different outcomes. Study participants had at least 12 weeks of follow-up. The study did not show significant differences between the efficacy of ACEIs and ARBs. Wang et al. published a systematic review and meta-analysis in 2018 examining the effects of ACEIs and ARBs on all-cause mortality and renal outcomes for diabetic patients with albuminuria [27]. The study participants were 10,378, and the outcome showed no reduction in all-cause mortality or cardiovascular events in patients treated with ACEIs and ARBs [27]. However, in a systematic review by lv et al. published in 2018, ACEIs were compared to ARBs in hypertensive patients with type 2 diabetes [28]. The study sampled 47,008 participants and revealed a significant reduction in all-cause mortality and major cardiovascular events by ACEIs. In contrast, ARBs failed to reduce all-cause mortality and did not reduce mortality due to cardiovascular events [28]. Chaugai et al. published a systematic review and meta-analysis in 2016 that compared the effects of RAS blockade in preventing atrial fibrillation [29]. The study examined 165,387 patients and showed a significant reduction in the onset and recurrence of atrial fibrillation but did not show any significant difference in the superiority of one class of the drug over another [29]. Overall, ACEIs appear to have higher efficacy in reducing all-cause mortality and mortality and morbidity due to cardiovascular events when compared to ARBs, as the studies that support this statement are more recent and have a significant number of study participants [6,9,28]. Although one of the studies that reported no significant difference in mortality and cardiovascular outcome between ARBs and ACEIs appears to have a very high number of participants (798,810), not all were assessed for the same outcome. The follow-up period appears short compared to other studies [11]. This is represented in Table 5.

**Table 5:** : Summary of ACEI vs ARB in outcomes of mortality and cardiovascular events ARB: Angiotensin receptor blocker; ACEI: angiotensin-converting enzyme inhibitor; DRI: direct renin inhibitor

Author and year of publication	Intervention/drug of study	Number of patients	Type of study	Result/conclusion
Salvador et al. (2017) [6]	Cardiovascular events, mortality (ACEI vs ARB)	73,761	Systematic review and meta-analysis	ACEI was shown to have higher efficacy in the reduction of cardiovascular events and all-cause mortality than ARB
Chaugai et al. (2016) [29]	Atrial fibrillation (ACEI vs ARB)	165,387	Systematic review and meta-analysis	Both ACEI and ARB showed similar efficacy in the reduction of atrial fibrillation
Xie et al. (2018) [9]	Cardiovascular events, mortality (blood pressure lowering drugs)	143,095	Systematic review and meta-analysis	ACEI was shown to reduce all-cause mortality and cardiovascular events, whereas ARB reduced the risk of combined events
Powers et al. (2012) [11]	Cardiovascular events, mortality (ACEI, ARB, and DRI)	798,810	Systematic review	No significant difference was found in efficacy between ACEI and ARB
Wang et al. (2018) [27]	Cardiovascular event, mortality (ACEI vs ARB in people with diabetes with albuminuria)	10378	Systematic review and meta-analysis	No reduction in all-cause mortality or cardiovascular events for either ACEI or ARB
lv et al. (2018) [28]	Cardiovascular event, mortality (ACEI vs ARB in patients with type 2 diabetes)	47,008	Systematic review and meta-analysis	ACEI showed a significant reduction in all-cause mortality and major cardiovascular events, whereas ARB did not

Adverse Effects

 A systematic review by Li et al. in 2014 compared ARBs to ACEIs in managing hypertension [2]. The study involved 11,007 patients with a follow-up period of at least one year. The study indicates that patients were slightly more likely to tolerate ARBs better than ACEIs. Patients taking ACEIs had a slightly higher chance of developing dry cough than ARBs, as established in the systematic review by Powers et al. in 2012 [11]. Angioedema has also been found to be more associated with ACEIs than ARBs. Heran et al. (2008) published a systematic review examining ARBs' efficacy in managing hypertension [30]. The study had 13,451 participants and revealed that when compared with placebo, ARBs were shown to reduce withdrawal due to adverse effects [30]. There was no change in withdrawal due to adverse effects when ACEIs were compared to placebo in a different systematic review by Heran et al. published in 2008 that reviewed the efficacy of ACEIs in lowering blood pressure in 12,954 participants [31]. Matcher et al. (2008) showed no significant difference in adverse effects when ACEIs and ARBs were compared when the rate of cough was excluded [12]. Overall, ARBs are better tolerated than ACEIs, likely due to the increased rate of dry cough associated with ACEIs [12]. This is summarized in Table 6.

**Table 6:** Summary of ACEI vs ARB in producing adverse reactions ARB: Angiotensin receptor blocker; ACEI: angiotensin-converting enzyme inhibitor; DRI: direct renin inhibitor

Author and year of publication	Intervention/drug of study	Number of patients	Type of study	Result/conclusion
Li et al. (2014) [2]	Adverse effects/(ACEI, ARB)	11,007	Systematic review	ARB is slightly better tolerated than ACEI
Powers et al. (2012) [11]	Adverse effects (ACEI, ARB, DRI)	798,810	Systematic review	ACEI has a stronger association with dry cough and angioedema than ARB
Matcher et al. (2008) [12]	Adverse effects (ACEI, ARB)	345,594	Systematic review	There was no significant difference in adverse effects between ACEI and ARB. However, there is a slight increase in the tolerability of ARB when compared to ACEI
Heran et al. (2008) [30]	Adverse effects (ARB)	13,451	Systematic review	Reduced withdrawal due to adverse effects when compared with placebo
Heran et al. (2008) [31]	Adverse effects (ACEI)	12,954	Systematic review	No change in withdrawal due to adverse effects when compared with placebo

Limitations of the Study

Our study has several limitations. Firstly, only studies published in English were selected for this study. Our study only examined systematic reviews and did not select other research articles. We excluded studies that included children. Only human studies were included. Some articles that met our inclusion and exclusion criteria were excluded from this study because there was no free access to the said articles. 

## Conclusions

This study was made to determine whether or not there is a difference between the use of ACEIs and ARBs in the treatment of hypertension. The study showed that both ACEIs and ARBs are similar in their efficacy in lowering blood pressure. However, ACEIs were shown to reduce all-cause mortality and mortality due to cardiovascular events. In contrast, ARBs have not demonstrated a similar outcome to ACEIs. More patients using AACEIs withdraw from the medication due to adverse effects than patients prescribed with ARBs.
